# Altered brain morphology and functional connectivity in postmenopausal women: automatic segmentation of whole-brain and thalamic subnuclei and resting-state fMRI

**DOI:** 10.18632/aging.205662

**Published:** 2024-03-23

**Authors:** Gwang-Won Kim, Kwangsung Park, Yun-Hyeon Kim, Gwang-Woo Jeong

**Affiliations:** 1 Advanced Institute of Aging Science, Chonnam National University, Gwangju 61186, Republic of Korea; 2 Department of Urology, Chonnam National University Hospital, Chonnam National University Medical School, Gwangju 61469, Republic of Korea; 3 Department of Radiology, Chonnam National University Hospital, Chonnam National University Medical School, Gwangju 61469, Republic of Korea

**Keywords:** brain morphology, functional connectivity, sex hormones, thalamic subnuclei

## Abstract

The transition to menopause is associated with various physiological changes, including alterations in brain structure and function. However, menopause-related structural and functional changes are poorly understood. The purpose of this study was not only to compare the brain volume changes between premenopausal and postmenopausal women, but also to evaluate the functional connectivity between the targeted brain regions associated with structural atrophy in postmenopausal women. Each 21 premenopausal and postmenopausal women underwent magnetic resonance imaging (MRI). T1-weighted MRI and resting-state functional MRI data were used to compare the brain volume and seed-based functional connectivity, respectively. In statistical analysis, multivariate analysis of variance, with age and whole brain volume as covariates, was used to evaluate surface areas and subcortical volumes between the two groups. Postmenopausal women showed significantly smaller cortical surface, especially in the left medial orbitofrontal cortex (mOFC), right superior temporal cortex, and right lateral orbitofrontal cortex, compared to premenopausal women (*p* < 0.05, Bonferroni-corrected) as well as significantly decreased functional connectivity between the left mOFC and the right thalamus was observed (*p* < 0.005, Monte-Carlo corrected). Although postmenopausal women did not show volume atrophy in the right thalamus, the volume of the right pulvinar anterior, which is one of the distinguished thalamic subnuclei, was significantly decreased (*p* < 0.05, Bonferroni-corrected). Taken together, our findings suggest that diminished brain volume and functional connectivity may be linked to menopause-related symptoms caused by the lower sex hormone levels.

## INTRODUCTION

Aging is a multifaceted and complex phenomenon characterized by physiological changes that exert a variety of effects on the structure and function of the brain [1]. One such pivotal transition in women is menopause, which encompasses the loss of ovarian reproductive function [2–4]. This transition, indicative of aging, leads to a decline in female sex hormones, such as estrogen. This decline has been linked to an elevated risk of neurodegenerative diseases, notably Alzheimer’s disease (AD) [5–7].

Estrogen plays a pivotal role in modulating neurotransmitter systems, neurotrophins, and brain cytoarchitecture [8–15]. These interactions might affect estrogen’s influence on neural systems governing mood and cognition. Notably, postmenopausal women have a higher risk of AD than men, and this sex difference risk has been hypothesized to be associated with the loss of estrogens following menopause [16]. The biological mechanisms underlying the increased AD risk in women are not fully understood [17]. Therefore, further studies are needed to evaluate the neurophysiological changes in postmenopausal women.

The investigation of menopause-related brain structural and functional alterations in women provides a unique opportunity to evaluate the early detection of neurodegenerative disease. Several studies [1, 6, 16, 18–20] have indicated a potential connection between menopause and brain volume, possibly mediated by the decrease in female sex hormones following menopause. For example, one structural magnetic resonance imaging (MRI) study [1] reported that postmenopausal women showed decreased gray matter volumes in the supplementary motor area, inferior frontal gyrus, olfactory cortex, and superior temporal gyrus compared to premenopausal women, which suggests that reduced volumes were closely associated with menopause-related symptoms. A similar study [20] demonstrated accelerated reduction of the hippocampal volume in postmenopausal women. Several morphometric studies [6, 16, 18, 19] focusing on the effects of estrogen therapy (ET) in postmenopausal women demonstrated enhanced cognitive function that are possibly associated with improvement of the corresponding brain structure and/or connectivity.

Consistent with the structural imaging findings, functional neuroimaging studies [21–23] reported that functional abnormalities in postmenopausal women were associated with cognitive impairment due to decreases in female hormone levels following menopause. A resting-state functional magnetic resonance imaging (fMRI) study [22] reported that early postmenopausal women exhibited significantly increased functional connectivity with the insula, prefrontal cortex, and superior frontal cortex compared to premenopausal women when the amygdala was used as the seed region and suggested that these regions were related to depressive states, poor sleep quality, and decreased executive function. Another study [21] concerning task-related connectivity suggested that postmenopausal women showed increased connectivity between the left and right hippocampi during the verbal encoding task compared to premenopausal women. These studies exclusively explored a specific brain region of interest (ROI), either the hippocampus or amygdala. However, a functional connectivity study using a seed region with reduced volume in postmenopausal women has not been published yet.

A recent MRI study [24] reported that thalamic volume loss was one of the first signs of cognitive decline in patients with early mild cognitive impairment (MCI). Cognitive decline is a frequent complaint during the transition to menopause in postmenopausal women [25]. The thalamus is an evolutionarily conserved structure with extensive reciprocal connections to cortical regions, and it plays an important role in learning and memory [26, 27]. A positron emission tomography (PET) study [28] reported that postmenopausal women receiving ET showed increased thalamic-basal ganglia connectivity compared to postmenopausal women without ET. To the best of our knowledge, no comparative neuroimaging study on alterations in the brain volume and functional connectivity, especially focusing on the thalamic subnuclei in premenopausal vs. postmenopausal women has been reported.

Thus, this study compared the brain volume changes in specialized with thalamic subnuclei, between the two groups, premenopausal and postmenopausal women. Also, the functional connectivity in targeted brain regions that are related with structural atrophy in postmenopausal women will be evaluated.

## MATERIALS AND METHODS

### Subjects

Twenty-one postmenopausal women (mean age: 55.3 ± 2.5 years) and 21 premenopausal women (mean age: 39.8 ± 7.6 years) underwent MRI. All women with right-handedness were recruited vis advertisements.

The premenopausal group was selected based on several criteria [1]: 1) absence of a menopause diagnosis as determined by the Stages of Reproductive Aging Workshop (STRAW) +10 criteria; and 2) regular menstrual bleeding. Ovulation day was estimated using the rhythm method. Participants with a history of psychiatric or neurological illnesses were excluded. Those who had undergone any hormonal or steroid treatment and those who had used oral contraceptives in the month preceding the study were also excluded. We included 21 premenopausal women in the premenstrual phase, 10 to 19 days prior to their expected period.

The postmenopausal group was selected based on the following inclusion criteria [1, 29]: 1) those with a confirmed diagnosis of menopause according to STRAW +10 criteria; and 2) an absence of menstrual bleeding for more than one year. Participants who had a history of a hysterectomy or bilateral oophorectomy were excluded. Those whose follicle-stimulating hormone (FSH) levels were less than 40 μg/mL were also excluded. Similar to the premenopausal group, these who had a history of neurological illnesses and those who had received hormonal or steroid treatments or used oral contraceptives in the month prior to the study were excluded. The average duration since the onset of menopause in these women was 4.8 ± 2.5 years. In terms of demographics, the majority of the premenopausal women, 18 out of 21, were married with an average of 1.8 ± 1.0 children, while 3 were single. In contrast, all postmenopausal women were married with an average of 2.8 ± 0.7 children.

This study was approved by the Institutional Review Board (IRB) of Chonnam National University Hospital (IRB-CNUH). The experimental procedure was explained to all Participants and written informed consent was obtained. All experimental procedures and methods were performed in accordance with relevant guidelines and regulations approved by the IRB-CNUH.

### Sex hormones

Levels of sex hormones including estradiol (E2), FSH, and luteinizing hormone (LH) were measured by chemiluminescent immunoassays using an ADVIA Centaur System (Bayer Healthcare, Chicago, IL, USA). Levels of estrogen, estriol (E3), free testosterone (free-T), and sex hormone-binding globulin (SHBG) were measured by radioimmunoassay using a gamma counter (Cobra 5010 Quantum, Packard Instrument Co, Meriden, CT, USA).

### MRI data acquisition

MRI and fMRI images were collected on a 3.0-T Magneton Tim Trio MR Scanner (Siemens Medical Solutions, Erlangen, Germany) with an 8-channel head coil. For structural imaging, T1-weighted sagittal images were acquired using a refined three-dimensional magnetization-prepared rapid-acquisition gradient echo (3D-MPRAGE) pulse sequence with the following parameters: a repetition time (TR) of 1900 ms and an echo time (TE) of 2.35 ms. The field of view (FOV) was maintained at 256 × 256 mm^2^ with a matrix size of 256 × 256. This protocol was designed to yield a comprehensive set of 176 slices per scan, with the number of excitations (NEX) set to 1.

Two 1-minute resting-state blood oxygen level-dependent (BOLD) scans were collected. These scans utilized a gradient echo-planar pulse sequence with the following parameters: a TR/TE of 2000 ms/30 ms, a flip angle of 90°, an FOV of 220 × 220 mm^2^, and a matrix size of 64 × 64. The slice gap was meticulously set to 0 mm to ensure contiguous coverage and optimal signal quality.

### Data processing and analysis

Anatomical (surface area and subcortical volume) and resting-state functional MR data were post-processed using FreeSurfer 6.0 software package (http://surfer.nmr.mgh.harvard.edu). Thalamic subnuclei were calculated using FreeSurfer 7.2 software package. Resting-state fMRI data analysis included runs with a temporal signal-to-noise ratio greater than 125 and a relative head motion value of less than 1 mm between one or more pairs of consecutive TRs [30].

### Structural analysis

T1-weighted images were analyzed with the procedure described in our previous studies [30, 31]. We incorporated advanced motion correction techniques and intensity non-uniformity adjustments. Talairach transformation was performed for each subject’s brain, followed by removal of non-brain tissues. We conducted a detailed segmentation for cortical gray matter, subcortical white matter, and deep gray matter volumetric structures [29, 30]. The cortical surface was reconstructed through triangular tessellation at the gray matter/white matter interface and the gray matter/cerebrospinal fluid boundary, followed by topological correction [29, 30].

**Figure 1:**
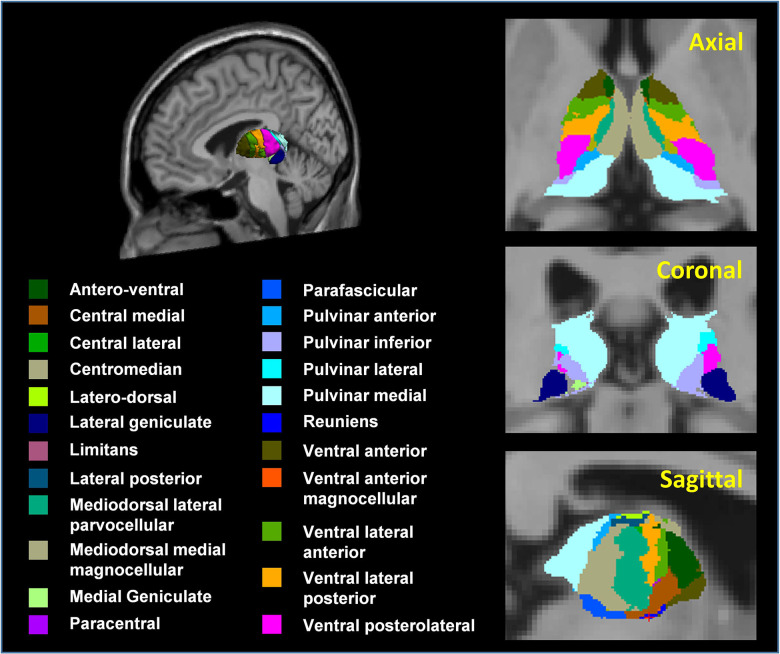
**3D atlas of the thalamus and its subnuclei segmentation.** Segmentation of thalamic subnuclei was performed using a built-in module of FreeSurfer.

Thirty brain regions of interest (ROIs) were selected from both hemispheres based on previous studies [1, 6, 19, 20, 32] focused on postmenopausal women, including the superior/middle/inferior frontal cortex, superior/middle/inferior temporal cortex, superior/inferior parietal lobule, lateral/medial orbitofrontal cortex, insula, hippocampus, thalamus, putamen, and amygdala. Twenty-three thalamic subnuclei (46 ROIs) were extracted for the left and right hemispheres, including the antero-ventral (AV), latero-dorsal (LD), lateral posterior (LP), ventral anterior (VA), ventral anterior magnocellular (VAmc), ventral lateral anterior (VLa), ventral lateral posterior (VLp), ventral posterolateral (VPL), central medial (CeM), central lateral (CL), paracentral (Pc), centromedian (CM), parafascicular (Pf), reuniens/medial ventral (MV-re), mediodorsal medial magnocellular (MDm), mediodorsal lateral parvocellular (MDl), lateral geniculate (LGN), medial geniculate (MGN), limitans/suprageniculate (L-SG), pulvinar anterior (PuA), pulvinar medial (PuM), pulvinar lateral (PuL), and pulvinar inferior (PuI) (Figure 1) [29].

### Resting-state fMRI analysis

Preprocessing of resting-state fMRI data was conducted using FS-FAST (the FreeSurfer Functional Analysis Stream) [30, 33]. First, functional data were realigned to correct for rigid head motion and corrected for slice time correction. These preprocessed functional data were then normalized to a common space (fsaverage). Normalized functional data were smoothed using a 3D Gaussian kernel (5-mm full-width-half-maximum). Spurious variance was reduced by nuisance regression derived from rigid body motion correction, signal regression in the white matter and cerebrospinal fluid, and global signal regression [30]. Functional data underwent a specialized low-pass filtering process at 0.08 Hz, effectively isolating neural signals from physiological-related noise. Based on reduced cortical surface areas in postmenopausal women, we conducted a voxel-wise resting-state connectivity analysis using the left medial orbitofrontal cortex (mOFC), right lateral OFC (lOFC), and right superior temporal cortex (STC) as seed regions. Resting-state functional connectivity between the seed region and the whole brain was determined via multiple regression analysis of the average time-series in each voxel [30].

### Statistical analyses

### Demographic characteristics and sex hormones

Independent samples t-test was used to compare the age and sex hormone levels of the premenopausal and postmenopausal women using SPSS (version 28.0, IBM, Armonk, NY, USA).

### Structural analysis

The Shapiro-Wilk test was used to assess the normality of the MR data. Multivariate analysis of variance, with age and whole brain volume as covariates, was used to compare cortical surface area and subcortical volume between the two groups. The significance level was set to 0.05 (*p* < 0.0017) after Bonferroni’s correction for 30 ROIs to adjust for multiple comparisons. Multivariate analysis of variance, with age and whole brain volume as covariates, was used to evaluate thalamic subnuclei volumes. The significance level was set to 0.05 (*p* = 0.001) after Bonferroni’s correction for 46 ROIs. A Spearman’s correlation test was used to evaluate the relationship between female sex hormone levels and surface area (or thalamic subnuclei volumes).

### Resting-state fMRI analysis

Multivariate analysis of variance, with age as a covariate, was used to compare functional connectivity between the two groups, which was corrected for multiple comparisons with Monte Carlo simulation using a voxel height threshold of p < 0.005 with a cluster-wise p-value (CWP) threshold of p < 0.05 [30]. A partial correlation adjusted for age was used to evaluate the relationship between left OFC-right thalamus functional connectivity and female sex hormone levels.

## RESULTS

### Age and serum sex hormone levels

There was a significant difference in age between the 2 groups (*p* < 0.001). Postmenopausal women had lower total estrogen (*p* < 0.001) and E2 levels (*p* < 0.001) and higher FSH (*p* < 0.001) and LH levels (*p* = 0.005) than premenopausal women (Table 1). However, there were no significant differences in E3, free-T, or SHBG between the two groups (*p* > 0.05, Bonferroni-corrected; Table 1).

**Table 1:** Sex hormone levels in premenopausal and postmenopausal women.

	**Premenopausal women (n=21)**	**Postmenopausal women (n=21)**	**p-value**
Estrogen^a^ (pg/mL)	566.8 ± 319.7	76.5 ± 40.9	< 0.001***
Estradiol (E2)^b^ (pg/mL)	212.7 ± 159.9	14.4 ± 7.6	< 0.001***
Estriol (E3)^c^ (pg/mL)	2.7 ± 1.4	2.3 ± 1.3	0.552
Free-testosterone^d^ (pg/mL)	0.4 ± 0.3	0.2 ± 0.1	0.294
Sex hormone binding^e^-globulin (SHBG, nmol/L)	102.0 ± 33.3	70.7 ± 17.3	0.026*
Follicle-stimulating^f^ hormone			
(FSH, mlU/mL)	6.1 ± 3.6	63.3 ± 21.5	< 0.001***
Luteinizing hormone^g^ (LH, mlU/mL)	14.2 ± 14.2	36.5 ± 11.5	0.005**

Data are presented as the mean ± standard deviation (SD).

*p < 0.05, **p < 0.01, ***p < 0.001.

Reference ranges for hormones in premenopausal and postmenopausal women (“Clinical Research Service” [GRQC-017 Rev.0] published in 2007 by Green Cross Reference Laboratory).

^a^Premenopausal women, more than 61 pg/mL; postmenopausal women, less than 60 pg/mL.

^b^Premenopausal women, 11 to 526 pg/mL; postmenopausal women, less than 37 pg/mL.

^c^Pregnant women, 49.2 to 375 ng/mL at 21 to 42 weeks.

^d^Women aged 20 to 39 years, 0.06 to 2.5 pg/mL; women aged 40 to 59 years, 0.04 to 2.0 pg/mL.

^e^Women, 16 to 120 nmol/L; men, 10 to 73 nmol/L.

^f^Premenopausal women, 1.5 to 33.4 mlU/mL; postmenopausal women, 23 to 116.3 mlU/mL.

^g^Premenopausal women, 0.5 to 73.6 mlU/mL; postmenopausal women, 15.9 to 54.0 mlU/mL.

### Cortical surface area and subcortical volume

**Figure 2:**
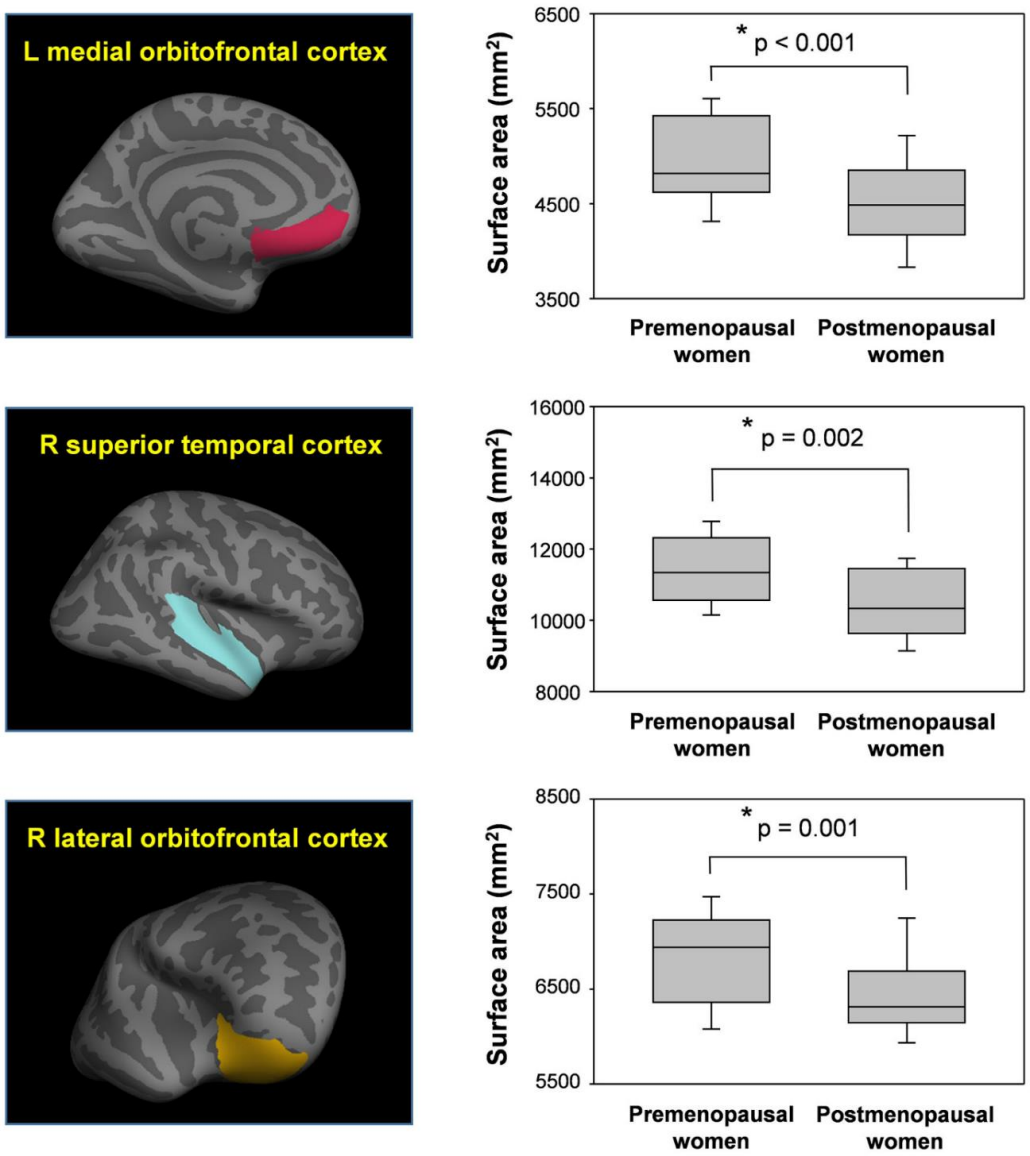
**Reduced surface areas in postmenopausal women compared to premenopausal women.** Postmenopausal women showed significantly reduced surface areas in the left medial orbitofrontal cortex (mOFC), right superior temporal cortex (STC), and right lateral orbitofrontal cortex (lOFC) compared to premenopausal women. *Meet Bonferroni-corrected significance level.

**Figure 3:**
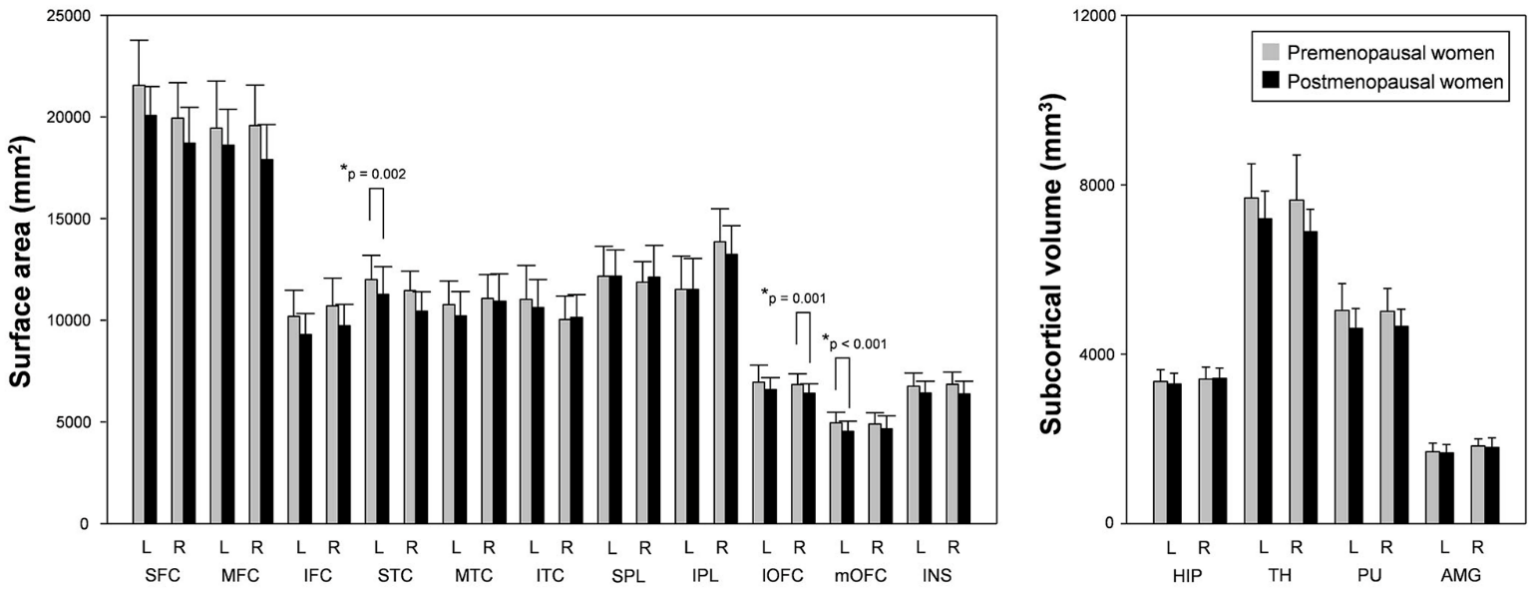
**Surface areas and subcortical volumes in postmenopausal women and premenopausal women.** Postmenopausal women showed significantly reduced surface areas of the left mOFC, right lOFC, and right STC compared to premenopausal women. L; left, R: right, SFC/MFC/IFC; superior/middle/inferior frontal cortex, STC/MTC/ITC; superior/middle/inferior temporal cortex, SPL/IPL; superior/inferior parietal lobule, lOFC/mOFC; lateral/medial orbitofrontal cortex, INS; insula, HIP; hippocampus, TH; thalamus, PU; putamen, AMG; amygdala. *Meet Bonferroni-corrected significance level.

As shown in Figures 2, 3, the surface area of the left mOFC, right lOFC, and right STC was significantly reduced in postmenopausal women compared to premenopausal women (*p* < 0.05, Bonferroni-corrected: Table 2). None of the other 27 ROIs were significantly different between the two groups (all *p* > 0.05; Figure 3 and Table 2).

**Table 2:** Comparison of brain volumes between premenopausal and postmenopausal women.

**Brain regions**		**Abbrev.**	**Premenopausal women (n = 21)**	**Postmenopausal women (n = 21)**	**F-value**	**P-value**	**Cohen’s d**	**Normality test (p-value)**
*a. Surface area*								
	L Superior frontal gyrus	SFC	21560.3 ± 2225.9	20069.9 ± 1428.8	10.8	0.002	1.04	0.116
	R Superior frontal gyrus		19950.4 ± 1735.1	18708.3 ± 1771.7	6.4	0.016	0.80	0.431
	L Middle frontal gyrus	MFC	19444.1 ± 2310.1	18616.8 ± 1763.5	5.2	0.028	0.72	0.853
	R Middle frontal gyrus		19578.4 ± 1991.2	17918.5 ± 1714.8	9.8	0.003	0.99	0.963
	L Inferior frontal gyrus	IFC	10190.0 ± 1287.9	9306.6 ± 1024.1	0.7	0.403	0.27	0.923
	R Inferior frontal gyrus		10709.5 ± 1366.6	9732.0 ± 1051.9	2.3	0.136	0.48	0.061
	L Superior temporal gyrus	STC	12008.4 ± 1188.2	11272.0 ± 1363.9	5.9	0.020	0.77	0.529
	R Superior temporal gyrus		11458.0 ± 960.1	10460.8 ± 955.2	11.5	0.002	1.07	0.423
	L Middle temporal gyrus	MTC	10768.6 ± 1174.2	10225.9 ± 1194.4	9.6	0.004	0.98	0.926
	R Middle temporal gyrus		11071.5 ± 1192.0	10936.0 ± 1360.6	7.5	0.009	0.87	0.470
	L Inferior temporal gyrus	ITC	11031.1 ± 1667.7	10630.0 ± 1377.7	8.0	0.007	0.89	0.007
	R Inferior temporal gyrus		10039.0 ± 1152.8	10145. ± 1113.9	1.1	0.301	0.33	0.709
	L Superior parietal lobule	SPL	12177.9 ± 1463.8	12172.3 ± 1303.0	0.6	0.452	0.25	0.857
	R Superior parietal lobule		11882.0 ± 1013.5	12115.9 ± 1569.5	0.7	0.405	0.27	0.863
	L Inferior parietal lobule	IPL	11520.5 ± 1642.0	11515.8 ± 1519.1	2.3	0.138	0.48	0.010
	R Inferior parietal lobule		13858.1 ± 1635.6	13232.4 ± 1428.5	2.7	0.110	0.52	0.210
	L Lateral orbitofrontal gyrus	lOFC	6969.0 ± 831.0	6596.4 ± 591.4	7.2	0.011	0.75	0.837
	R Lateral orbitofrontal gyrus		6843.4 ± 525.4	6426.9 ± 450.6	12.5	0.001	1.12	0.131
	L Medial orbitofrontal gyrus	mOFC	4972.3 ± 503.5	4540.3 ± 496.2	14.6	< 0.001*	0.21	0.412
	R Medial orbitofrontal gyrus		4898.9 ± 550.9	4670.1 ± 624.7	5.1	0.030	0.71	0.014
	L Insula	INS	6748.6 ± 647.5	6434.1 ± 580.0	4.7	0.037	0.69	0.889
	R Insula		6856.9 ± 597.0	6384.4 ± 623.8	5.1	0.030	0.71	0.020
*b. Subcortical volume*								
	L Hippocampus	HIP	3362.8 ± 277.5	3297.8 ± 249.0	2.4	0.130	0.49	0.988
	R Hippocampus		3411.9 ± 284.4	3431.7 ± 236.9	0.5	0.479	0.22	0.375
	L Thalamus	TH	7691.5 ± 804.2	7196.5 ± 655.6	1.8	0.192	0.42	0.096
	R Thalamus		7637.2 ± 1065.0	6892.8 ± 529.4	6.0	0.019	0.78	0.012
	L Putamen	PU	5037.3 ± 626.8	4611.9 ± 466.8	1.7	0.204	0.41	0.788
	R Putamen		5013.4 ± 544.3	4651.1 ± 413.6	5.5	0.025	0.74	0.347
	L Amygdala	AMG	1695.3 ± 206.9	1672.9 ± 192.4	1.1	0.310	0.33	0.013
	R Amygdala		1834.4 ± 169.6	1804.0 ± 223.9	6.0	0.019	0.78	0.478

Multivariate analysis of variance, with age and whole brain volume as covariates, was used to compare surface size and subcortical volume between the two groups. Postmenopausal women showed significantly reduced surface areas in the left mOFG, right lOFG, and right STG compared to premenopausal women (*p* < 0.05, Bonferroni-corrected). Normality was evaluated by the Shapiro-Wilk test. L; left, R; right, abbrev; abbreviation.

*Meet Bonferroni-corrected significance level.

**Figure 4:**
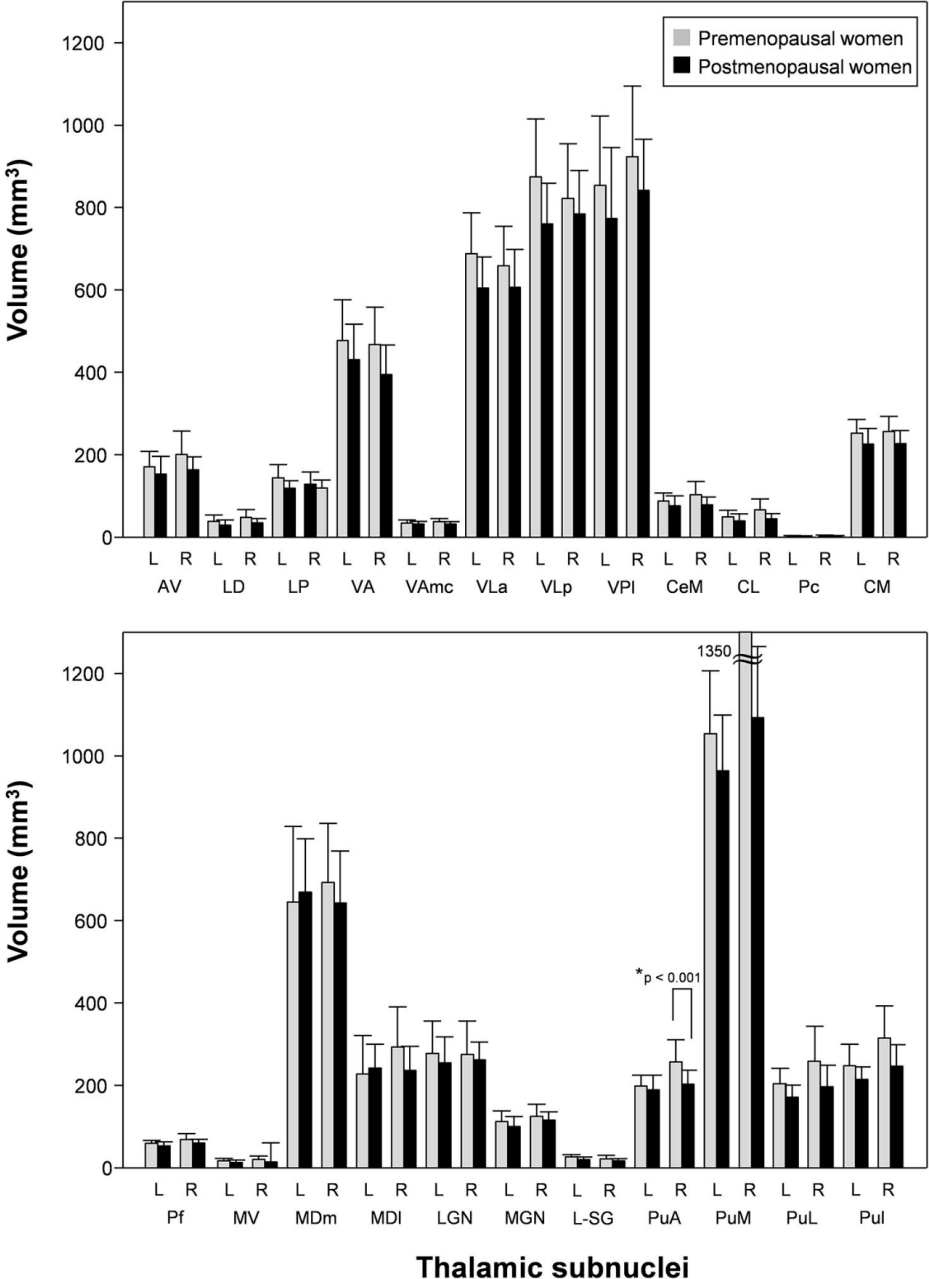
**Thalamic subnuclei volumes in postmenopausal vs. premenopausal women.** Postmenopausal women showed significantly reduced volume of the right PuA compared to premenopausal women. L; left, R: right, AV; antero-ventral, LD; latero-dorsal, LP; lateral posterior, VA; ventral anterior, VAmc; ventral anterior magnocellular, VLa; ventral lateral anterior, VLp; ventral lateral posterior, VPL; ventral posterolateral, CeM; central medial, CL; central lateral, Pc; paracentral, CM; centromedian, Pf; parafascicular, MV-re; reuniens (medial ventral), MDm; mediodorsal medial magnocellular, MDl; mediodorsal lateral parvocellular, LGN; lateral geniculate, MGN; medial geniculate, L-SG; limitans (suprageniculate), PuA; pulvinar anterior, PuM; pulvinar medial, PuL; pulvinar lateral, PuI; pulvinar inferior. *Meet Bonferroni-corrected significance level.

Although postmenopausal women did not show volume atrophy in the right thalamus, the volume of the right PuA, which is one of thalamic subnuclei, was significantly decreased (*p* < 0.05, Bonferroni-corrected; Figure 4 and Table 3). There was also a significant volume difference in the right PuA between the two groups (Mann-Whitney U-test; *p* < 0.001). None of the other 45 ROIs were significantly different between the two groups (all *p* > 0.05; Figure 4 and Table 3).

**Table 3:** Comparison of thalamic subnuclei volumes between premenopausal and postmenopausal women.

**Thalamic nuclei**		**Abbrev.**	**Premenopausal women**	**Postmenopausal women**	**F-value**	**P-value**	**Cohen’s d**	**Normality test (p-value)**
Anterior	L Anteroventral	AV	171.7 ± 37.7	153.5 ± 43.4	0.3	0.564	0.17	0.976
	R Anteroventral		201.6 ± 56.9	163.7 ± 32.1	2.4	0.128	0.49	0.217
Lateral	L Laterodorsal	LD	39.1 ± 14.9	30.2 ± 12.0	0.1	0.787	0.10	0.694
	R Laterodorsal		48.9 ± 19.0	36.0 ± 10.0	0.3	0.602	0.17	0.465
	L Laterodorsal posterior	LP	144.8 ± 31.8	118.9 ± 18.8	8.9	0.005	0.94	< 0.001
	R Laterodorsal posterior		129.0 ± 30.1	119.9 ± 19.9	0.0	0.960	0.00	0.988
Ventral	L Ventral anterior	VA	478.4 ± 98.5	431.3 ± 86.4	0.1	0.783	0.10	0.191
	R Ventral anterior		468.2 ± 90.6	394.9 ± 72.2	4.4	0.043	0.66	0.448
	L Ventral anterior magnocellular	VAmc	35.5 ± 6.8	32.7 ± 6.3	0.2	0.681	0.14	0.250
	R Ventral anterior magnocellular		38.4 ± 7.6	33.0 ± 5.8	2.8	0.100	0.53	0.108
	L Ventral lateral anterior	VLa	688.9 ± 99.2	605.5 ± 75.0	8.5	0.006	0.92	0.026
	R Ventral lateral anterior		659.6 ± 95.8	607.1 ± 91.8	2.9	0.099	0.54	0.174
	L Ventral lateral posterior	VLp	875.4 ± 140.5	761.0 ± 98.6	10.7	0.002	1.03	0.043
	R Ventral lateral posterior		823.3 ± 132.5	785.3 ± 105.6	2.0	0.165	0.45	0.643
	L Ventral posterolateral	VPL	854.6 ± 168.0	774.2 ± 172.3	3.0	0.090	0.55	0.226
	R Ventral posterolateral		924.1 ± 171.7	842.8 ± 124.0	2.4	0.130	0.49	0.961
Intralaminar	L Central medial	CeM	87.9 ± 20.0	76.4 ± 24.4	0.1	0.712	0.10	0.724
	R Central medial		104.0 ± 32.0	79.3 ± 18.8	3.3	0.076	0.57	0.032
	L Central lateral	CL	50.0 ± 16.0	40.6 ± 16.7	0.0	0.850	0.00	0.548
	R Central lateral		67.2 ± 26.8	45.3 ± 12.8	1.7	0.198	0.41	0.009
	L Paracentral	Pc	4.0 ± 0.6	3.6 ± 0.8	0.9	0.347	0.30	0.044
	R Paracentral		4.9 ± 1.0	4.2 ± 0.5	2.8	0.101	0.53	0.008
	L Centromedian	CM	253.3 ± 33.2	227.1 ± 37.8	2.4	0.128	0.49	0.300
	R Centromedian		256.9 ± 37.2	227.5 ± 32.1	3.1	0.086	0.56	0.468
	L Parafascicular	Pf	60.8 ± 7.0	54.4 ± 10.4	2.8	0.104	0.53	0.612
	R Parafascicular		70.6 ± 13.8	61.3 ± 9.7	2.3	0.139	0.48	0.016
Medial	L Reuniens (medial ventral)	MV-re	18.5 ± 5.8	14.2 ± 5.7	0.4	0.543	0.20	0.293
	R Reuniens (medial ventral)		22.3 ± 8.1	15.1 ± 47	2.9	0.097	0.54	0.018
	L Mediodorsal medial magnocellular	MDm	645.8 ± 184.5	670.0 ± 130.2	3.2	0.081	0.57	< 0.001
	R Mediodorsal medial magnocellular		694.2 ± 143.4	644.5 ± 125.7	9.5	0.004	0.98	0.680
	L Mediodorsal lateral parvocellular	MDl	228.7 ± 93.5	243.5 ± 58.0	3.9	0.056	0.62	< 0.001
	R Mediodorsal lateral parvocellular		295.0 ± 96.9	237.6 ± 58.6	10.7	0.002	1.03	0.170
Posterior	L Lateral geniculate	LGN	279.3 ± 78.1	256.5 ± 62.9	0.3	0.567	0.17	0.496
	R Lateral geniculate		276.6 ± 81.2	263.7 ± 42.9	0.2	0.653	0.14	0.361
	L Medial Geniculate	MGN	113.5 ± 26.1	101.4 ± 24.1	0.9	0.358	0.30	0.370
	R Medial Geniculate		126.5 ± 29.3	117.1 ± 20.0	1.1	0.299	0.33	0.901
	L Limitans (suprageniculate)	L-SG	28.4 ± 5.2	21.1 ± 6.2	8.3	0.006	0.91	0.768
	R Limitans (suprageniculate)		23.3 ± 8.3	18.5 ± 5.4	0.8	0.390	0.28	0.004
	L Pulvinar anterior	PuA	199.7 ± 26.6	190.9 ± 35.2	0.4	0.508	0.20	0.215
	R Pulvinar anterior		258.1 ± 54.4	204.4 ± 34.1	16.2	< 0.001*	1.27	0.029
	L Pulvinar medial	PuM	1055.3 ± 152.6	964.3 ± 135.9	0.0	0.885	0.00	0.187
	R Pulvinar medial		1350.8 ± 272.4	1094.0 ± 172.5	8.7	0.005	0.93	0.025
	L Pulvinar lateral	PuL	205.8 ± 36.8	172.5 ± 29.9	4.7	0.036	0.69	0.306
	R Pulvinar lateral		260.0 ± 84.8	198.4 ± 52.5	5.7	0.022	0.76	0.008
	L Pulvinar inferior	PuI	249.2 ± 52.2	216.3 ± 30.2	0.1	0.780	0.10	0.136
	R Pulvinar inferior		316.3 ± 78.4	248.3 ± 52.2	2.6	0.118	0.51	0.069

Multivariate analysis of variance, with age and whole brain volume as covariates, was used to compare thalamic subnuclei volumes between the two groups. Postmenopausal women showed significantly reduced volume in the right PuA compared to premenopausal women (*p* < 0.05, Bonferroni-corrected). Normality was evaluated by the Shapiro-Wilk test. L; left, R; right, abbrev; abbreviation. *Meet Bonferroni-corrected significance level.

### Correlation between structural changes and sex hormone levels

**Figure 5:**
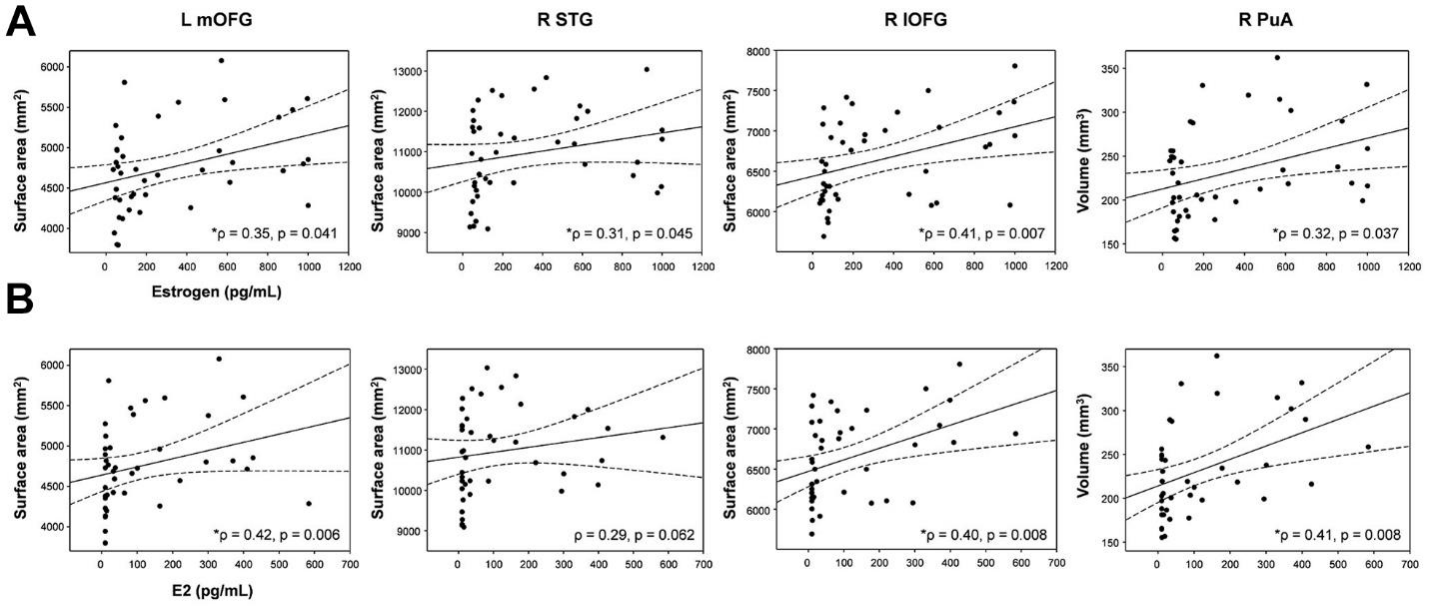
**Correlations between brain structural changes and female sex hormone levels.** (**A**) Estrogen levels were positively correlated with the surface areas of the left mOFC (ρ = 0.35, p = 0.041), right STG (ρ = 0.31, p = 0.045), and right lOFG (ρ = 0.41, p = 0.007) and the volume of the right PuA (ρ = 0.32, p = 0.037), respectively. (**B**) E2 levels were positively correlated with the surface area of the left mOFC (ρ = 0.42, p = 0.006) and right lOFC (ρ = 0.40, p = 0.008) and the volume of the right PuA (ρ = 0.41, p = 0.008), respectively. The dotted lines show 95% confidence intervals. L; left, R; right, mOFC; medial orbitofrontal cortex, STC; superior temporal cortex, lOFC; lateral orbitofrontal cortex, PuA; pulvinar anterior.

Estrogen levels were positively correlated with the surface area of the left mOFC (*p* = 0.35, *p* = 0.041), right STG (*p* = 0.31, *p* = 0.045), and right lOFG (*p* = 0.41, *p* = 0.007) and the volume of the right PuA (*p* = 0.32, *p* = 0.037), respectively (Figure 5). E2 levels were positively correlated with the surface area of the left mOFC (*p* = 0.42, *p* = 0.006) and right lOFC (*p* = 0.40, *p* = 0.008) and the volume of the right PuA (*p* = 0.41, *p* = 0.008), respectively (Figure 5). Note that no correlation between the E2 level and the surface area of the right STG was observed.

### Functional connectivity analysis

**Figure 6:**
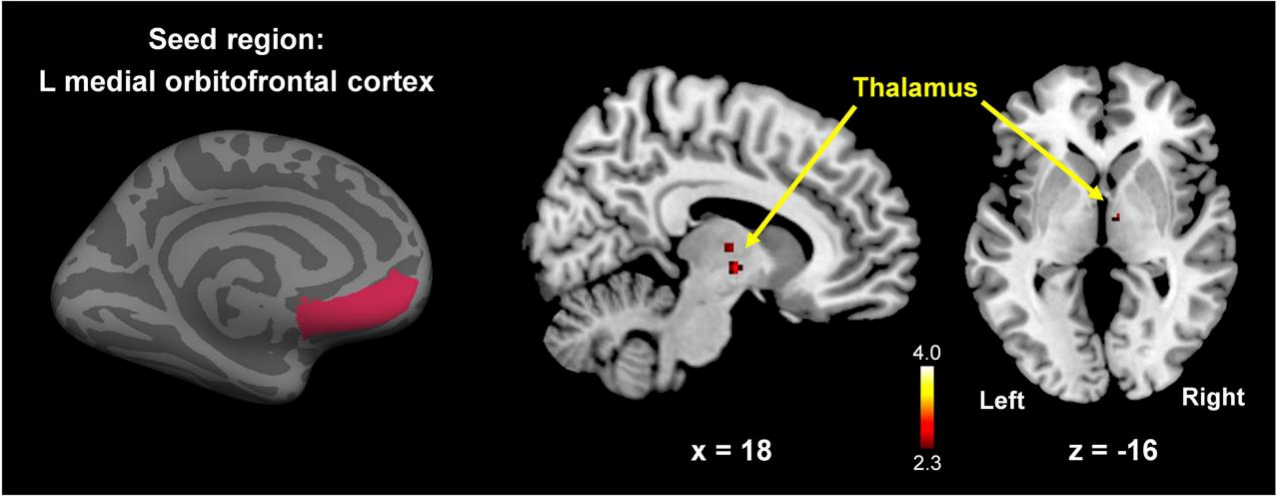
**Between-group comparison map (premenopausal women vs. postmenopausal women) of the left mOFC functional connectivity.** Postmenopausal women showed significantly lower functional connectivity between the left mOFC and right thalamus compared to premenopausal women (*p* < 0.005, Monte-Carlo corrected). The color-coded pixels were scaled to the range (t-value) more than the cut-off threshold (*p* < 0.005).

Significantly lower functional connectivity between the left mOFC and right thalamus was found in postmenopausal women compared to premenopausal women (*p* < 0.005, Monte-Carlo corrected; Figure 6). However, no significant difference (*p* > 0.005) was detected in whole-brain connectivity of the other two ROIs (right lOFC or right STC) between the two groups.

## DISCUSSION

### Summary of main findings

In this study, postmenopausal women exhibited lower total estrogen and E2 levels and higher FSH and LH levels compared to premenopausal women. In the structural analysis, the surface areas of the left mOFC, right lOFC, and right STC were significantly lower in postmenopausal women compared to premenopausal women. In addition, the functional connectivity between the left mOFC and the thalamus in postmenopausal women was significantly lower than premenopausal women. Also, the volume of the right PuA, which is one of the important thalamic subnuclei, was significantly reduced in postmenopausal women compared to premenopausal women (Figure 4). Given that decreases in female sex hormones are considered as a sign of menopause [2, 9, 34], our findings suggest that diminished mOFC size in postmenopausal women is closely associated with the correspondent functional abnormalities.

### Reduced surface areas in postmenopausal women

Postmenopausal women showed significantly lower levels of surface areas in the left mOFC and right lOFC compared to premenopausal women. The OFC is implicated in multiple cognitive processes, including inhibitory control, context memory, recency judgment, and behavior choice [35]. Decreases in female sex hormones following menopause may be linked to the left mOFC and right lOFC, further supporting the hypothesis that menopause-related hormonal changes can lead to alterations in brain morphology and cognitive dysfunction [10, 19]. The effects of estrogen on the structure of the prefrontal cortex, including the OFC, are relevant to the kinds of cognitive symptoms reported by women undergoing natural, medical, and surgical menopause [8, 32, 34, 36, 37]. The PFC has a relatively high density of estrogen receptors, and estrogen receptor mRNA is also highly expressed throughout the PFC [38–40]. A previous study [40] found a positive correlation between E2 levels and cortical thickness in the orbitofrontal cortex. In our study, the surface area of the left mOFC was positively correlated with estrogen and E2 levels, and the surface area of the right lOFC was positively correlated with estrogen levels. A morphometric study [6] on ET found increased volume in the OFC of postmenopausal women receiving ET compared to postmenopausal women. A similar study [32] reported significantly lower gray matter concentrations in the bilateral orbitofrontal cortex of postmenopausal women receiving ET than in postmenopausal women without ET.

We found lower surface areas in the right STC of postmenopausal women than in premenopausal women. This finding is consistent with prior evidence reporting that the STC volume was positively correlated with female sex hormones [1]. The STC has been implicated in language processing and social perception and is widely recognized as being sensitive to estrogen changes [1, 37, 38]. Estrogen exposure was positively correlated with metabolism in the STC [41, 42]. Concerning the close connection between the estrogen level and STC volume, our findings support a potential role of decreases in sex hormones following menopause due to the correspondent brain structural atrophy. However, further study is needed to elucidate the specific cognitive and emotional implications in connection with these structural changes.

### Functional connectivity between mOFC and thalamus

Together with the surface area analysis, we analysis revealed significantly decreased functional connectivity between the left mOFC and right thalamus in postmenopausal women compared to premenopausal women. This finding reinforces the hypothesis that the left orbitofrontal-bilateral thalamus connectivity is associated with cognitive impairment [43]. Several studies [44–46] demonstrated key roles for orbitofrontal-thalamic connections in the occurrence and development of psychiatric symptoms and cognitive impairment. In addition, OFC has long been known to play a central role in female sex hormones. For example, a study [47] reported that activation of the OFC from sexually relevant stimuli was positively correlated with the estradiol-to-progesterone ratio in women. Also, other studies [8, 48] demonstrated positive correlations between ET and brain activity in the OFC during emotion identification tasks and negative emotional image presentations, which suggests a potential link between the OFC and female sex hormones. Diminished OFC size and functional connectivity in postmenopausal women may be associated with cognitive dysfunction caused by reduced sex hormone levels in connection with neurophysiological change following menopause.

### Decreased thalamic subnuclei volume

Although postmenopausal women did not show volume atrophy in the right thalamus, the volume of right PuA, which is one of thalamic subnuclei, was significantly decreased. In addition, the volume of the right PuA was positively correlated with E2 levels. Although the association between cognitive function and estrogen in postmenopausal women is well known, little is known about thalamic subnuclei volumes. The thalamus, with its cortical and subcortical connections, is a critical node in networks supporting cognitive functions known to decline in normal aging [49]. Conversion from MCI to AD dementia was associated with reduced gray matter volume in the right thalamus, suggesting an association with worse cognitive performance [24]. Decreased left mOFC-right thalamus functional connectivity in postmenopausal women may affect specific thalamic subnuclei volumes, such as the PuA. Specifically, the pulvinar is a thalamic nucleus, which has long been considered a key structure for sensory processing and attention [50]. There is another evidence on morphological change of the PuA in patients with attention-deficit disorder [51]. Decreased PuA volume in postmenopausal women is closely related to decreases in female sex hormone levels following menopause. Our findings provide novel insight into the structural and functional changes in the brain associated with menopause.

### Limitations and future directions

This study had some limitations. Firstly, our sample size was relatively small, with only 21 postmenopausal women included in the study. This small sample size may limit the generalizability of our findings and potentially introduce bias. To overcome this limitation, we used multiple-comparison correction to analyze brain volumes between the two groups. Secondly, our study groups were not matched for age, probably bringing about age-related volume alterations. Our findings should be interpreted cautiously because of the limitations related to age differences and the statistical methods used. Future studies should consider matching participants for age to control the potential effects of aging on brain volume and functional connectivity. Lastly, our study did not include a cognitive test to assess the cognitive abilities of the subjects. Given that menopause and the associated decreases in sex hormone levels have been linked to cognitive decline, the inclusion of cognitive testing would provide a more comprehensive understanding of the relationship between menopause, brain structure and function, and cognitive ability. Future research should incorporate cognitive assessments to elucidate the potential cognitive implications of the observed structural and functional brain changes in postmenopausal women.

## CONCLUSIONS

Postmenopausal women showed significantly lower left mOFC, right lOFC, and right STC surface areas, reduced right PuA volume, and decreased left mOFC-right thalamus functional connectivity compared to premenopausal women. If replicated in an independent sample, these findings will be helpful for understanding the effects of menopause on the altered brain volume and functional connectivity in postmenopausal women.
